# Molybdenum Enzymes and How They Support Virulence in Pathogenic Bacteria

**DOI:** 10.3389/fmicb.2020.615860

**Published:** 2020-12-11

**Authors:** Qifeng Zhong, Bostjan Kobe, Ulrike Kappler

**Affiliations:** ^1^Australian Infectious Diseases Research Centre, School of Chemistry and Molecular Biosciences, The University of Queensland, St. Lucia, QLD, Australia; ^2^Institute for Molecular Bioscience, The University of Queensland, St. Lucia, QLD, Australia

**Keywords:** infectious disease, metabolic pathways, pyranopterin molybdenum enzymes, bacterial pathogen, molybdenum enzymes, virulence, energy generation, metalloenzyme

## Abstract

Mononuclear molybdoenzymes are highly versatile catalysts that occur in organisms in all domains of life, where they mediate essential cellular functions such as energy generation and detoxification reactions. Molybdoenzymes are particularly abundant in bacteria, where over 50 distinct types of enzymes have been identified to date. In bacterial pathogens, all aspects of molybdoenzyme biology such as molybdate uptake, cofactor biosynthesis, and function of the enzymes themselves, have been shown to affect fitness in the host as well as virulence. Although current studies are mostly focused on a few key pathogens such as *Escherichia coli, Salmonella enterica, Campylobacter jejuni*, and *Mycobacterium tuberculosis*, some common themes for the function and adaptation of the molybdoenzymes to pathogen environmental niches are emerging. Firstly, for many of these enzymes, their role is in supporting bacterial energy generation; and the corresponding pathogen fitness and virulence defects appear to arise from a suboptimally poised metabolic network. Secondly, all substrates converted by virulence-relevant bacterial Mo enzymes belong to classes known to be generated in the host either during inflammation or as part of the host signaling network, with some enzyme groups showing adaptation to the increased conversion of such substrates. Lastly, a specific adaptation to bacterial in-host survival is an emerging link between the regulation of molybdoenzyme expression in bacterial pathogens and the presence of immune system-generated reactive oxygen species. The prevalence of molybdoenzymes in key bacterial pathogens including ESKAPE pathogens, paired with the mounting evidence of their central roles in bacterial fitness during infection, suggest that they could be important future drug targets.

## Introduction

Molybdenum and tungsten are redox-active transition metals and, when incorporated into enzymes, are able to mediate a variety of reactions including the oxidation of substrates using oxygen from water (Murray et al., 1966; Hille, 2005; Schwarz et al., 2009; Hille et al., 2014; Hagen, 2017). Currently, the so-called mononuclear Mo/W enzymes, which contain a single Mo or W atom complexed by an organic pyranopterin (PPT) cofactor at their active site, are the most abundant group of Mo/W-containing enzymes (Magalon et al., 2011; Iobbi-Nivol and Leimkühler, 2013; Hille et al., 2014; Rothery and Weiner, 2015; Hagen, 2017). Mo/W enzymes are evolutionarily old and likely already existed in the last universal common ancestor (Lebrun et al., 2003; Zhang and Gladyshev, 2008; Schoepp-Cothenet et al., 2012; Nitschke and Russell, 2013; Mayr et al., 2020; Wells et al., 2020). As a result, they are found in all domains of life and are particularly abundant in bacteria, where more than 50 distinct types of enzymes have been identified (Leimkühler and Iobbi-Nivol, 2016; Maia et al., 2017), and physiological functions range from sulfur compound oxidation, degradation of aromatic compounds to supporting anaerobic respiration in bacteria (Hille et al., 2014; Maia et al., 2017).

Bacterial Mo/W enzymes are found in each of the three major Mo/W enzyme families, the xanthine oxidase (XO) and sulfite oxidase (SO) families, which also contain enzymes from eukaryotic organisms, and the dimethyl sulfoxide (DMSO) reductase (DMSOR) enzyme family, which exclusively contains prokaryotic enzymes. Very few bacteria lack mononuclear Mo/W enzymes, and this is usually associated with highly adapted lifestyles that are found in Chlamydiae and certain types of Firmicutes (Zhang and Gladyshev, 2008).

**Figure 1:**
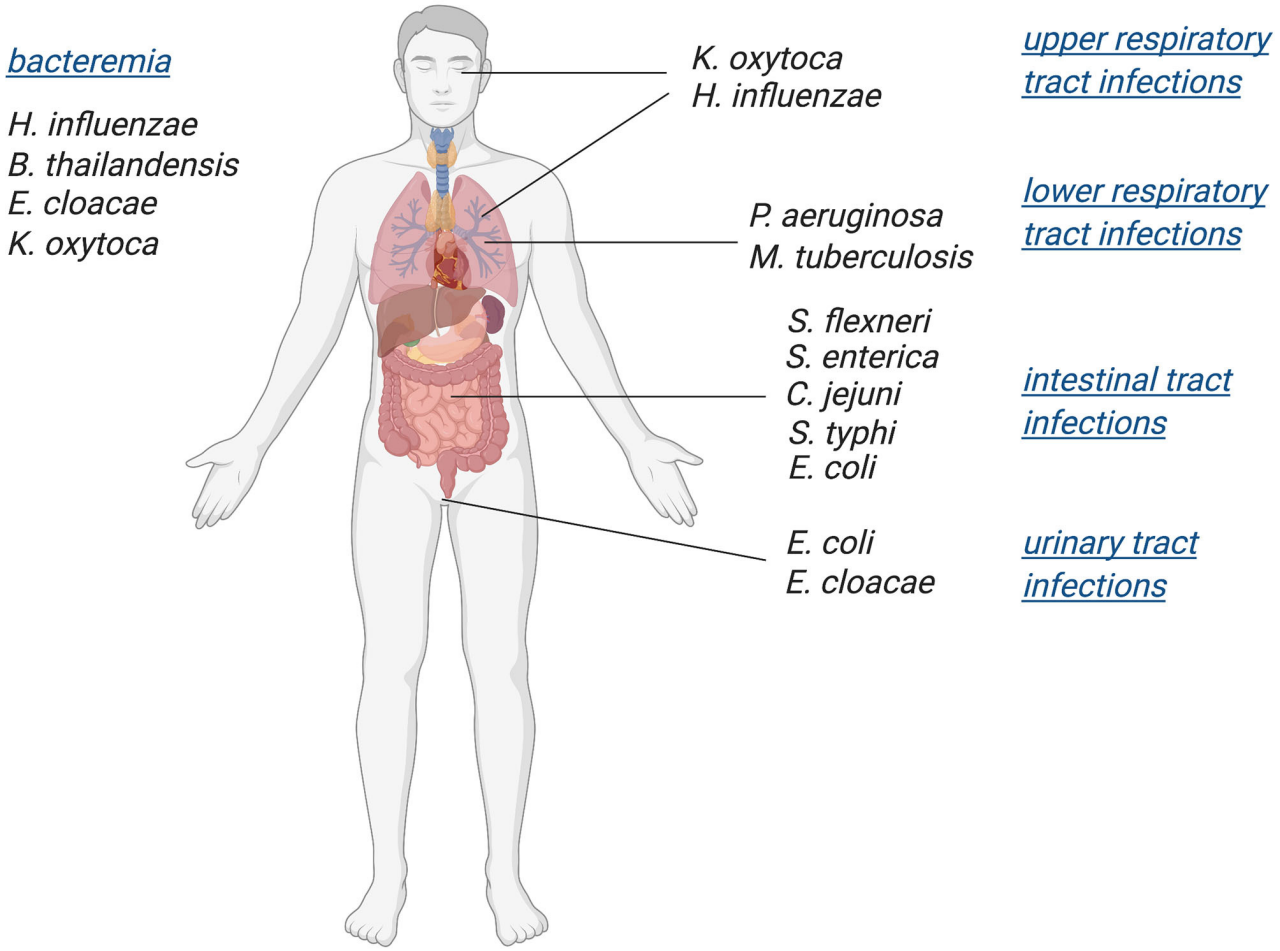
Schematic overview of bacterial pathogens in which Mo-enzymes have been shown to support virulence. Infections with these pathogens affect a variety of body niches, as well as systemic infections.

While mononuclear Mo/W enzymes have been studied for over five decades, the role of these enzymes in pathogenic bacteria and their influence on bacterial fitness and virulence has only recently become an area of focus. Various studies have linked in-host survival of prevalent pathogenic bacteria such as *Mycobacterium tuberculosis (Mtb), Escherichia coli*, and *Salmonella enterica* to the presence of functional molybdoenzymes (Winter et al., 2010, 2013b; Denkel et al., 2013; Williams et al., 2014). Here, we have collated current data on the roles of Mo enzymes as well as enzymes involved in molybdenum uptake and PPT cofactor biosynthesis in bacterial virulence, revealing that Mo enzymes are essential for both body niche specific and systemic infections (Figure 1). Since tungstoenzymes are predominantly found in extremophiles (Andreesen and Makdessi, 2008; Hagen, 2017; Crichton, 2019; Niks and Hille, 2019; Seelmann et al., 2020), the focus of this review will be on molybdenum-containing enzymes.

## Molybdenum Uptake, Cofactor Biosynthesis, and Bacterial Virulence

Two processes are required for the synthesis of Mo-PPT cofactors, molybdate uptake via a transport protein and the actual cofactor biosynthesis pathway, and defects in either process have profound impacts on Mo enzyme activity.

### Molybdenum Uptake

In most bacteria, the ATP-binding-cassette (ABC)-type molybdate transport system, ModABC, mediates high affinity uptake of molybdate from the environment (Rech et al., 1995; Hu et al., 1997; Imperial et al., 1998; Lawson et al., 1998; Self et al., 2001; Gisin et al., 2010; Perinet et al., 2016; Xia et al., 2018). The substrate-binding protein of this transporter, ModA, is a molybdate-specific, type II periplasmic-binding-protein (PBP2, cl21456) that interacts with the ModB and ModC proteins that form the channel for molybdate import and hydrolyze ATP to provide the energy required for transport, respectively (Hu et al., 1997; Lawson et al., 1998; Self et al., 2001; Santacruz et al., 2006; Hollenstein et al., 2007; Davidson et al., 2008). In the absence of ModABC, non-specific (e.g., sulfate transporters) or lower affinity Mo uptake systems such as the MolBCA transporter found in some strains of *Haemophilus influenzae* may enable uptake of small amounts of molybdate (Tirado-Lee et al., 2011; Rice et al., 2014). Defects in the Mo uptake system will lower the availability of molybdate for incorporation into Mo enzymes and result in a reduction or even loss of Mo enzyme activities, which can reduce bacterial environmental fitness. High affinity uptake of molybdate is particularly important for pathogenic bacteria, as they may be competing with the host for molybdate. Molybdate concentrations are known to be <1 μg of molybdenum per 1 g of wet organ/ash in many body niches affected by pathogenic bacteria such as the GI tract, lung, bladder, and uterus (Schroeder et al., 1970).

ModABC has been identified as essential for host interactions in *Salmonella typhi, Mtb*, and *Pseudomonas aeruginosa*, where strains defective in molybdate uptake were attenuated in different models of infection such as macrophages and mice (Table 1) (Contreras et al., 1997; Camacho et al., 1999; Perinet et al., 2016). A *P. aeruginosa* PAO1 Δ*modA* mutant also showed significantly reduced biofilm formation as a result of an unidentified physiological change (Pederick et al., 2014), however, biofilm phenotypes were not reported for Δ*modA* mutants in other bacteria. Loss of high affinity Mo uptake also caused reduced nitrate reductase activity or impaired growth of *E. coli, P. aeruginosa, S. typhi*, and *Campylobacter jejuni* on nitrate-containing media (Glaser and DeMoss, 1971; Contreras et al., 1997; Taveirne et al., 2009; Pederick et al., 2014), which likely resulted from the reduced availability of molybdate for incorporation into the molybdenum-dependent nitrate reductase active site.

**Table 1:** Defects in molybdate uptake or Mo-PPT biosynthesis genes and their effects on bacterial virulence.

**Pathway involved**	**Gene(s)**	**Organism**	**Mutant type**	**Host interaction model(s) used**	**Effect on virulence**	**References**
Mo uptake	*modA*	*Mycobacterium tuberculosis*	Transposon mutant	Mouse lung infection	++	Camacho et al., 1999
		*Pseudomonas aeruginosa*	Isogenic mutant	*In vitro* biofilm formation	++	Pederick et al., 2014
		*Pseudomonas aeruginosa*	Transposon mutant	Mouse lung infection	++	Perinet et al., 2016
	*modC*	*Salmonella typhi*	Transposon mutant	Human epithelial cells	++	Contreras et al., 1997
Mo-PPT synthesis	*moaA*	*Enterobacter cloacae*	Isogenic mutant	Mouse colitis model	++	Hughes et al., 2017
		*Klebsiella oxytoca*	Isogenic mutant	Mouse colitis model	++	Hughes et al., 2017
		*Mycobacterium tuberculosis*	Isogenic mutant	Mouse colitis model	++	Winter et al., 2013b; Hughes et al., 2017
		*Salmonella typhi*	Transposon mutant	Human epithelial cells	++	Contreras et al., 1997
	*moaA1-D1* locus	*Mycobacterium tuberculosis*	Isogenic mutant	Mouse necrotic lesion (hypoxic)	++	Levillain et al., 2017
	*moaC1*	*Mycobacterium tuberculosis*	Transposon mutant	Primate lung infection	++	Dutta et al., 2010
	*moaC1* and *moaD1*	*Mycobacterium tuberculosis*	Transposon mutant	Macrophage phagosome maturation	++	Brodin et al., 2010
	*moaC1* and *moaX*	*Mycobacterium tuberculosis*	Transposon mutant	Macrophage infection	++	Rosas-Magallanes et al., 2007
	*moeA*	*Burkholderia thailandensis*	Transposon mutant	*In vitro* biofilm formation	++	Andreae et al., 2014
	*moeB1*	*Mycobacterium tuberculosis*	Transposon mutant	Macrophage infection	++	MacGurn and Cox, 2007
	*PA1006* (*tusA* homolog)	*Pseudomonas aeruginosa*	Isogenic mutant	Burned mouse model; rat lung infection	++	Filiatrault et al., 2013
Mo-bisPGD synthesis	*mobAB*	*Escherichia coli*	Transposon mutant	Mouse and duck systemic infection	++	Zhang et al., 2019
	*mobA*	*Haemophilus influenzae*	Isogenic mutant	Human epithelial cells and neutrophils; mouse lung infection	+^a^	Dhouib et al., 2015
	*mobA*	*Mycobacterium tuberculosis*	Isogenic mutant	Human monocytes; mouse and guinea pig lungs infection	+^b^	Williams et al., 2015

*++strong reduction; + weak reduction*.

a *Only a defect in mouse colonization*.

b *Only a defect in persistence in guinea pig lungs*.

The significant reduction in bacterial virulence in the absence of effective Mo uptake has led to the molybdate-binding protein ModA being proposed as a promising drug target. Alignments of known ModA amino acid sequences, however, show that there is a relatively high divergence between species (sequence identities: 20–64%), and additionally there is some variability in the molybdate binding residues, which could make it difficult to find drugs that bind effectively to ModA from a variety of bacteria (Hu et al., 1997; Lawson et al., 1998; Santacruz et al., 2006; Hollenstein et al., 2007).

### The Molybdenum-Pyranopterin Cofactor and Its Biosynthesis

**Figure 2:**
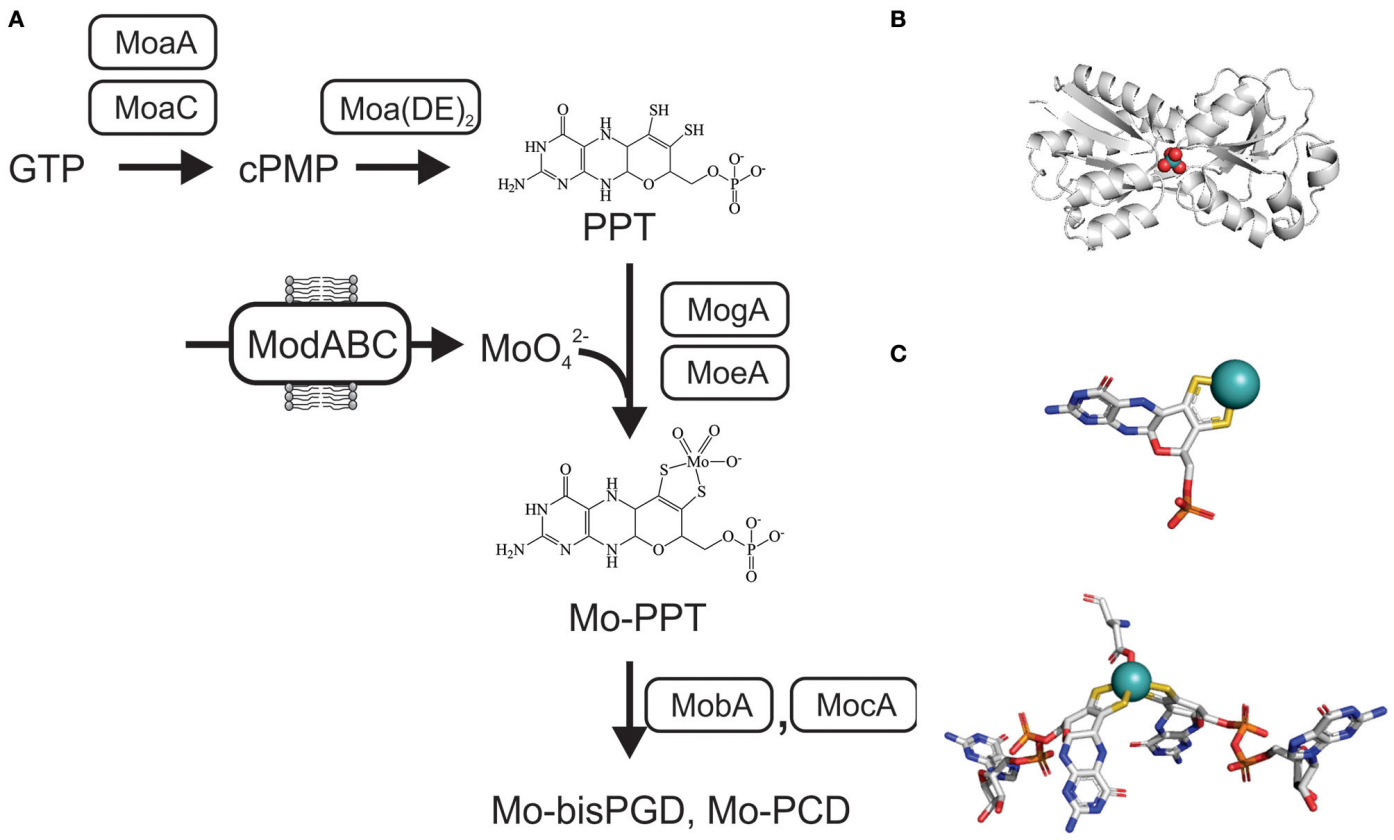
Schematic representation of the biosynthesis of Mo-PPT and Mo-PPT-dinucleotide forms from GTP/CTP in bacteria. cPMP, cyclic pyranopterin monophosphate; PPT, pyranopterin; PGD/PCD, pyranopterin guanine/cytosine dinucleotide; (MoaDE)_2_, PPT synthase. **(A)** Overview over molybdate uptake and Mo-PPT biosynthesis **(B)** Crystal structure of ModA from *Escherichia* (PDB: 1AMF) with a molybdate ion (${\text{MoO}}_{4}^{-}$) bound, bluewhite- protein backbone, teal– molybdenum, red– oxygen. **(C)** 3D conformations of Mo-PPT and Mo-bisPGD in the enzyme-bound form, Mo-PPT is from MsrP (PDB: 1XDQ), Mo-bisPGD – NarGH (PDB: 1R27). Colors: white – carbon, blue – nitrogen, orange – phosphor, red – oxygen, yellow – sulfur, light teal– molybdenum.

The tricyclic pyranopterin cofactor (PPT, previously referred to as molybdopterin, MPT) that complexes the Mo atom is required for activity of all mononuclear molybdoenzymes. The Mo-PPT cofactor is synthesized from GTP in three major steps that involve six highly conserved enzymes, and at least two accessory enzyme complexes (Leimkühler et al., 2011; Mendel, 2013; Hille et al., 2014; Leimkühler, 2014; 2020; Mayr et al., 2020) (Figure 2). Depending on the molybdoenzyme family an enzyme belongs to, the basic Mo-PPT may contain different modifications (Magalon et al., 2011; Hille et al., 2014; Leimkühler, 2020).

Initially, cyclization of GTP via carbon rearrangement, catalyzed by MoaA and MoaC, leads to the formation of a cyclic pyranopterin monophosphate (cPMP) (Wuebbens et al., 2000; Hanzelmann and Schindelin, 2004; Hover et al., 2013, 2015; Srivastava et al., 2013; Yokoyama and Lilla, 2018; Pang et al., 2020). Sulfuration of cPMP, the second step, is carried out by PPT synthase, a heterotetrametric (α_2_β_2_) complex consisting of the MoaD and MoaE proteins, and leads to the formation of a PPT molecule (Pitterle et al., 1993; Daniels et al., 2008) (Figure 2). The PPT synthase reaction is supported by three accessory enzymes, MoeB, IscS, and TusA, which resulfurate the MoaD subunit of PPT synthase after each reaction cycle using cysteine as the sulfur donor (Leimkühler and Rajagopalan, 2001; Leimkühler et al., 2001; Zhang et al., 2010; Dahl et al., 2013; Kozmin et al., 2013; Mayr et al., 2020) (Figure 2).

The third and final step of Mo-PPT biosynthesis is the insertion of the Mo atom. This requires activation of the PPT by adenylation, which is catalyzed by MogA (Liu et al., 2000), and is followed by MoeA-mediated Mo incorporation (Schrag et al., 2001; Xiang et al., 2001; Nichols and Rajagopalan, 2005) (Figure 2). While Mo-PPT is found in most plant and mammalian Mo enzymes, many bacterial Mo enzymes require a modified, dinucleotide version of the Mo-PPT, such as pyranopterin guanine/cytosine dinucleotide (PGD/PCD) and, less frequently, also an adenine dinucleotide (Borner et al., 1991; Rajagopalan and Johnson, 1992; Guse et al., 2003; Neumann et al., 2009b). Formation of these Mo-PPT derivatives is catalyzed by the MocA and MobA proteins, with MobB likely to contribute to the process (Palmer et al., 1994, 1996; Guse et al., 2003; Neumann et al., 2009b; Leimkühler, 2014) (Figure 2). Pathways for the incorporation of the cofactors into individual molybdoenzymes follow enzyme-specific pathways that often require maturation chaperones (Leimkühler and Klipp, 1999; Oresnik et al., 2001; Turner et al., 2004; Iobbi-Nivol and Leimkühler, 2013; Cherak and Turner, 2017). Further information about Mo-PPT biosynthesis can be found in several excellent review papers (Leimkühler et al., 2011; Magalon et al., 2011; Iobbi-Nivol and Leimkühler, 2013; Mendel, 2013; Hille et al., 2014; Leimkühler and Iobbi-Nivol, 2016; Leimkühler, 2020; Mayr et al., 2020).

Mo-PPT biosynthesis enzymes have been shown to be crucial for virulence in several pathogenic bacteria, including *E. coli* and *Mtb* (Table 1) (MacGurn and Cox, 2007; Rosas-Magallanes et al., 2007; Brodin et al., 2010; Dutta et al., 2010; Winter et al., 2013b; Williams et al., 2014, 2015; Hughes et al., 2017; Levillain et al., 2017). The essential role of many of these Mo-PPT synthesis defects in bacterial pathogens was not identified by targeted mutagenesis, but instead during global transposon mutagenesis aimed at identifying genes essential for host-pathogen interactions (Contreras et al., 1997; Camacho et al., 1999; MacGurn and Cox, 2007; Rosas-Magallanes et al., 2007; Brodin et al., 2010; Dutta et al., 2010; Perinet et al., 2016; Zhang et al., 2019). As a result, mechanistic insights into the physiological effects of these defects are often unavailable.

As a general rule, mutations in the Mo-PPT synthesis pathway completely abolish cofactor formation, leading to a loss of activity for all Mo enzymes present and creating a pleiotropic phenotype. This is clearly demonstrated by studies in *Mtb* and pathogens of the family Enterobacteriaceae, where regardless of which Mo-PPT-biosynthetic enzyme was lost, the mutant strains displayed significantly reduced fitness and/or virulence in different host-pathogen interaction models (Table 1) (Contreras et al., 1997; Rosas-Magallanes et al., 2007; Brodin et al., 2010; Dutta et al., 2010; Winter et al., 2013b; Hughes et al., 2017; Levillain et al., 2017).

Virulence attenuation was also associated with defects in accessory proteins of Mo-PPT synthesis, such as the PPT synthase resulfuration complex in *Mtb* and *P. aeruginosa* (Table 1) (MacGurn and Cox, 2007; Filiatrault et al., 2013). A *P. aeruginosa* strain lacking the TusA-related PA1006 protein, which is part of the resulfurase complex (Kozmin et al., 2013), exhibited an ~10-fold reduction in cytoplasmic and membrane fraction molybdate concentrations, as well as impaired biofilm formation and maturation *in vitro* (Filiatrault et al., 2013; Tombline et al., 2013). PA1006 was also shown to interact with almost all Mo-PPT biosynthesis proteins (Tombline et al., 2013), providing a possible reason for the observed defects and introducing a new layer of complexity in the Mo-PPT cofactor biosynthesis pathway.

By contrast, mutations in enzymes catalyzing the formation of Mo-PPT dinucleotide forms have less striking effect on bacterial virulence (Table 1), possibly because they only affect enzymes requiring specific types of PPT cofactors. Mutations in *mobA*, which encodes the enzyme responsible for Mo-bisPGD formation (Lake et al., 2000), decreased the activities of all DMSOR family molybdoenzymes in *P. aeruginosa, Mtb*, and *H. influenzae* (Noriega et al., 2005; Tombline et al., 2013; Dhouib et al., 2015; Williams et al., 2015), but only resulted in a mild reduction of bacterial fitness in the *Mtb* and *H. influenzae* mutants *in vivo* (Dhouib et al., 2015; Williams et al., 2015). However, an *E. coli* carrying mutations in both *mobA* and *mobB* genes [the product of latter was proposed to have a non-essential role in Mo-PPT cofactor synthesis (Iobbi-Nivol et al., 1995; Leimkühler, 2014, 2020)], showed clearly reduced virulence in both mouse and duck systemic infection models (Zhang et al., 2019). This suggests that further investigations are needed to elucidate role of MobA-MobB complexes in the maturation of Mo enzymes from different enzyme families.

## Xanthine Oxidase Family Enzymes and Their Roles in Bacterial Pathogens

**Figure 3:**
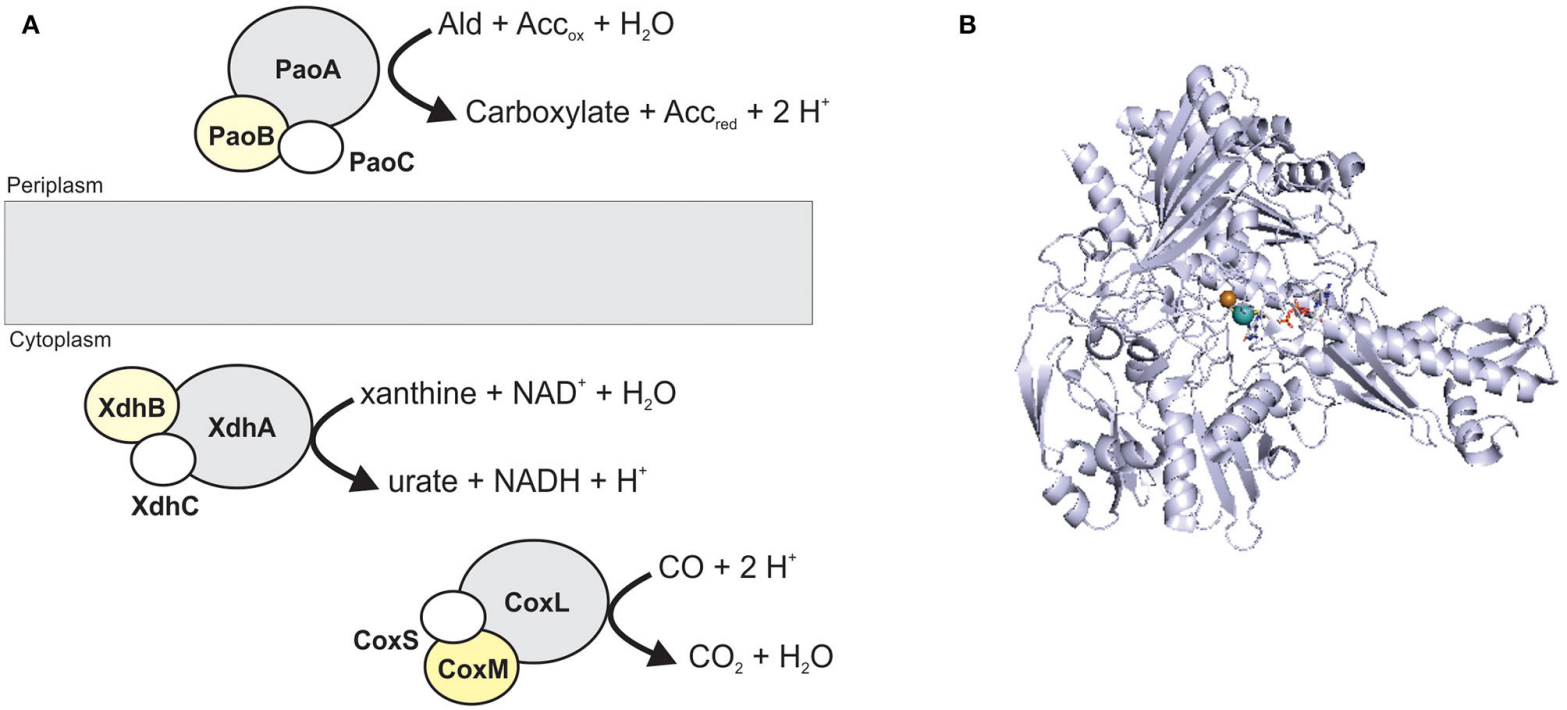
The structure and location of typical XO family enzymes. **(A)** schematic representation of enzyme structure. Enzymes of this family most commonly have a heterotrimeric (αβγ) structure containing a Mo-binding catalytic subunit (gray); an iron-sulfur protein (white); a FAD-binding subunit (yellow). CoxMSL is a carbon monoxide dehydrogenase, XdhABC is a xanthine dehydrogenase and PaoABC is an aldehyde oxidoreductase. **(B)** Crystal structure of CoxL, the catalytic subunit of the carbon monoxide dehydrogenase from *Oligotropha carboxidovorans* (PDB: 1N63) with the CuMo-PCD cofactor shown in stick form. Colors: white – carbon, blue – nitrogen, orange – phosphor, red – oxygen, yellow – sulfur, light teal– molybdenum, brown– copper, protein backbone - bluewhite.

XO family enzymes typically catalyze conversions of nucleobases and aldehydes, with typical representatives being xanthine oxidoreductases (XORs, include both XOs and xanthine dehydrogenases, XDHs), aldehyde oxidases (AOX), and carbon monoxide dehydrogenases (CODHs) (Hille et al., 2014; Nishino et al., 2017). The majority of enzymes found in this family are cytoplasmic proteins, and only some, such as particular bacterial aldehyde oxidases (Neumann et al., 2009a), are located in the bacterial periplasm (Figure 3).

AOXs oxidize numerous aromatic and non-aromatic aldehydes to their respective carboxylic acids, and are responsible for metabolizing various xenobiotics and drugs in mammals (Romão et al., 2017; Terao et al., 2020), while the bacterial aldehyde oxidoreductases are likely involved in metabolizing aromatic aldehydes for detoxification (Correia et al., 2016).

XO family enzymes typically contain not only a molybdenum redox center, but also at least one iron-sulfur cluster, while flavin adenine dinucleotide (FAD) is not always present (Bray et al., 1996; Nishino et al., 2017). In eukaryotic as well as some bacterial enzymes, two or more cofactor binding domains may be fused to form a single polypeptide, and enzymes with between one and three subunits have been identified (Figure 3) (Gremer and Meyer, 1996; Leimkühler et al., 1998; Enroth et al., 2000; Neumann et al., 2009a; Coelho et al., 2012; Otrelo-Cardoso et al., 2014). The active site of XO enzymes is typically pentacoordinated, with two Mo-PPT dithiolene sulfurs, an oxo-, a hydroxo/hydro-, and a sulfido-ligand coordinating the Mo atom. The sulfido-group can be replaced by selenium or another oxo-group in some XO enzymes, and interestingly, there is no amino acid ligand to the Mo center (Boyington et al., 1997; Schräder et al., 1999; Hille et al., 2014; Nishino et al., 2017). All mammalian enzymes of this family contain Mo-PPT, while Mo-PCD is present in most bacterial enzymes (Gremer and Meyer, 1996; Leimkühler et al., 1998; Magalon et al., 2011; Hille et al., 2014; Leimkühler, 2020; Terao et al., 2020).

A large number of studies have reported properties of XO family enzymes in bacteria, where they support the use of CO or purines as carbon, and in the latter case, also nitrogen sources as well as the detoxification of aldehydes (Leimkühler et al., 1998; Santiago et al., 1999; Xi et al., 2000; Park et al., 2003; Neumann et al., 2009a), however, only limited insights are available on their role in bacterial pathogens. The reactions catalyzed by XO family enzymes are relevant during infection as potential substrate molecules occur in the host, and XO family enzymes are found in human pathogens, including several opportunistic pathogens and species on the WHO top priority pathogen list such as *Acinetobacter baumannii, Mtb*, and Enterobacteriaceae family species (World Health Organization, 2017).

### Xanthine Dehydrogenases

XORs catalyze the conversion of the purine base hypoxanthine to xanthine, and xanthine to uric acid, which is part of purine catabolism (Schwarz and Belaidi, 2013; Wang et al., 2016; Nishino et al., 2017). Human XOR deficiency leads to xanthinuria, which is characterized by elevated blood xanthine levels, and, if untreated, can eventually result in critical conditions such as urinary tract calculi or renal failure (Schwarz and Belaidi, 2013; Battelli et al., 2016). Human XOR is highly expressed in the liver and small intestine (Sarnesto et al., 1996), implying that xanthine is present in these body niches. In bacteria, XDHs are often required for growth on purine compounds as nitrogen and/or possibly also carbon sources, again indicating a role in purine catabolism (Christiansen et al., 1997; Xi et al., 2000).

XDH occurs in bacteria present in the human gut microbiome, and there is evidence that XDH may contribute to the overall fitness of these bacteria by mediating access to (hypo-)xanthine as an additional nitrogen or carbon source (Christiansen et al., 1997; Xi et al., 2000). *B. subtilis* growth with xanthine or hypoxanthine as the sole nitrogen source was XDH-dependent and could be inhibited by allopurinol (Christiansen et al., 1997). In contrast, growth of *E. coli* on a (hypo-)xanthine-supplemented medium required an additional nitrogen source such as aspartate, suggesting that purines might be used as a carbon source in *E. coli* (Xi et al., 2000). However, to the best of our knowledge, there is so far no direct evidence for XDHs or AOXs having a role in bacterial virulence.

### Carbon Monoxide (CO) Dehydrogenases

Carbon monoxide dehydrogenases (CODH, CoxMSL) (Figure 3) catalyze the oxidation of CO to CO_2_ and enable growth with CO as the sole energy and carbon source in carboxydobacteria (Santiago et al., 1999; King and Weber, 2007; Robb and Techtmann, 2018). CODHs contain a special Mo-Cu binuclear active site (Dobbek et al., 2002; Gnida et al., 2003) and have been found in several pathogenic bacteria including *P. aeruginosa, Burkholderia* spp., and *Mycobacterium* spp. (King and Weber, 2007).

In the human body, CO is produced by the action of heme oxygenases (*Hmox1*) and can act as a signaling molecule in general physiological responses such as blood pressure adjustment, and in immune cells such as macrophages, where it activates the NALP3 inflammasome for bacterial clearance (Chung et al., 2008; Chin and Otterbein, 2009; Wegiel et al., 2014). CO also acts directly as an antimicrobial, attributable to its ability to bind metalloproteins and alter their biological functions (Nobre et al., 2007; Zacharia and Shiloh, 2012).

Resistance of *Mtb* to CO produced by macrophages has been attributed to the presence of the CutBCA CODH, an enzyme that is also present in many other *Mycobacterium* spp. (Shiloh et al., 2008; Song et al., 2010; Zacharia and Shiloh, 2012). CO triggers expression of the *Mtb* dormancy regulon (Kumar et al., 2008; Shiloh et al., 2008), and high levels (20.5 U/mg) of CODH activity were present in *Mtb* cultures following *in vitro* growth in the presence of CO (Park et al., 2007). CutBCA not only allows *Mtb* to use CO as a carbon source, but also possesses nitric oxide dehydrogenase activity (Park et al., 2007), which is thought to be unique to mycobacterial CODHs as these enzymes are phylogenetically distinct from other bacterial MoCu-CODHs (Song et al., 2010). In keeping with this, purified mycobacterial CODH was able to protect *E. coli* from nitrosative stress (Park et al., 2007), and reduced transcription of the *cut* operon in *Mycobacterium* sp. strain JC1 increased susceptibility to NO stress (Lee et al., 2017). However, direct evidence for an *in vivo* role of the *Mtb* CODH in host-association is still needed.

## Sulfite Oxidase Family Enzymes in Pathogens

Sulfite oxidase (SO) family enzymes are found in all domains of life and, in addition to prokaryotic and eukaryotic sulfite-oxidizing enzymes (SOEs), this enzyme family also includes plant assimilatory nitrate reductases (aNRs), as well as bacterial sulfur dehydrogenases, sulfoxide reductases and a group of uncharacterized, single-subunit, cytoplasmic enzymes (Kappler, 2008; Workun et al., 2008; Kappler and Enemark, 2015). A new class of human Mo enzymes, the mitochondrial amidoxime reducing components (mARCs), have also been tentatively assigned to this enzyme family in preliminary investigations (Wahl et al., 2010; Llamas et al., 2017; Kubitza et al., 2018).

**Figure 4:**
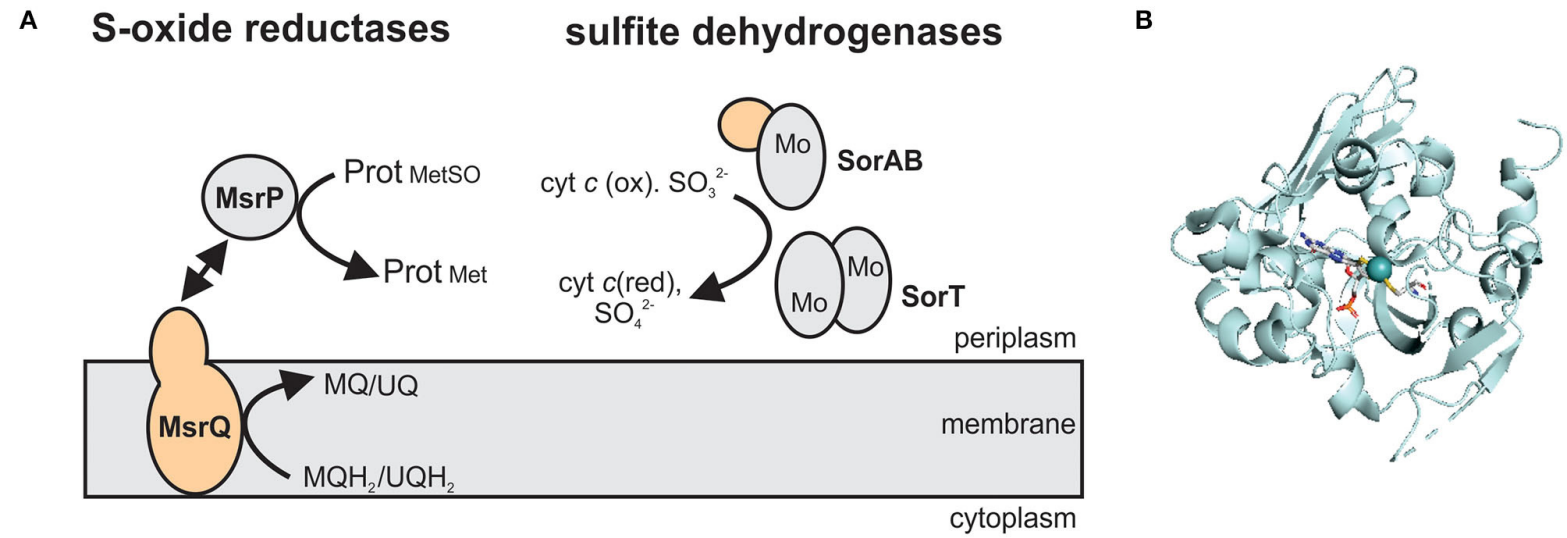
The structure and subcellular location of SO family enzymes associated with bacterial virulence. **(A)** Overview of enzyme structures. SorAB and SorT are two types of sulfite dehydrogenases that oxidize sulfite to sulfate, while the MsrP protein reduces protein-bound methionine sulfoxides with electrons transferred from the membrane integral cytochrome MsrQ. Gray – Mo-binding subunit; orange – heme-binding subunits. MQ – menaquinone; MQH_2_ - menaquinol; UQ – ubiquinone; UQH_2_ – ubiquinol. **(B)** Crystal structure of the methionine sulfoxide reductase MsrP from *Escherichia coli* (PDB: 1XDQ) with the Mo-PPT cofactor shown in stick form. Colors: white – carbon, blue – nitrogen, orange – phosphor, red – oxygen, yellow – sulfur, light teal – molybdenum, protein backbone – pale-cyan.

SO family enzymes typically contain a Mo-PPT cofactor bound to a protein with a characteristic sulfite oxidase (SUOX)-fold (Figure 4). The Mo atom is complexed by the two dithiolene sulfurs of the PPT cofactor as well as a cysteine residue and two oxo ligands, one of which is active in catalysis (Workun et al., 2008; Kappler and Enemark, 2015). Unlike the XO family enzymes that contain Fe/S clusters and, frequently, also FAD as redox cofactors, SO family enzymes often contain only a Mo center, or a combination of the Mo redox center with one or more heme groups, while FAD is only present in plant aNRs (Crawford et al., 1988; Kappler and Schwarz, 2017).

At least three enzyme subgroups that differ in the structure and size of the catalytic Mo subunit and the amino acid residues surrounding the active site have been identified in this enzyme family (Figure 4) (Kappler, 2008), namely (1) YedY and YedY-like enzymes that are prevalent in bacterial pathogens, (2) the main SOE group that also contains plant aNRs and bacterial sulfur dehydrogenases, and (3) uncharacterized, single subunit enzymes from environmental bacteria and archaea (Figure 4). For further information on the structural and catalytic properties of this family of enzymes, the reader is referred to recent review articles (Kappler, 2008, 2011; Hille et al., 2014; Kappler and Enemark, 2015; Kappler and Schwarz, 2017).

### YedY and YedY-Like Enzymes

Genes encoding the group 1 SO family enzymes are predominantly found in bacterial pathogens and form two subgroups, the YedY (group 1A) and YedY-like (group 1B) enzymes (Kappler, 2008). The two subgroups differ in the size of the Mo-binding domain (1A: 35 kDa, 1B: 30 kDa) and the cytochrome they interact with (Kappler, 2008). The membrane-bound cytochrome subunits these enzymes interact with provide electrons from the quinone pool to the catalytic subunit of the enzyme (Loschi et al., 2004; Brokx et al., 2005) (Figure 4).

Group 1 SO family enzymes likely all act as reductases: the currently best characterized enzyme of this group is the *E. coli* MsrP (formerly YedY) methionine sulfoxide reductase that belongs to group 1A which also contains sequences from *Yersinia* spp. and *C. jejuni* (Kappler, 2008). MsrP/YedY was initially characterized as a weak DMSO reductase, before further investigations revealed that this enzyme is a methionine sulfoxide reductase, able to repair both free methionine sulfoxide (MetSO) and MetSO residues in oxidatively damaged periplasmic proteins (Loschi et al., 2004; Gennaris et al., 2015). Two *E. coli* cell envelope proteins, SurA, an outer membrane protein chaperone, and Pal, a lipoprotein that mediates outer membrane integrity, have been identified as physiological substrates of MsrP (Gennaris et al., 2015). At present, MsrP is the only Mo-containing sulfoxide reductase identified outside of the DMSOR enzyme family, to which these enzymes normally belong (Gennaris et al., 2015). Interestingly, expression of the *msrPQ* genes is induced by hypochlorite, an antimicrobial compound produced by neutrophils during infection, and regulated by the YedY/W two-component system (Gennaris et al., 2015), which clearly links this enzyme to host-associated environmental conditions; however, infection studies confirming this have not been reported so far.

While an *E. coli* Δ*msrP* strain has not been tested in infection models, an MsrP-related protein from *C. jejuni* encoded by the *cj0379c* gene (NCBI Gene ID: 904702) was shown to be required for successful colonization of the chicken gut (Hitchcock et al., 2010). Chickens are the natural host of *C. jejuni*, a bacterium that causes gastroenteritis in humans when ingested.

The Cj0379 protein is also a group 1A enzyme and, like *E. coli* MsrP, displays poor S-oxide reductase activity with standard substrates (Hitchcock et al., 2010). However, a Δ*cj0379c* strain had a higher sensitivity to nitrosative stress but not superoxides, and a slower NO degradation rate *in vitro* (Hitchcock et al., 2010), suggesting that Cj0379 has a role in nitrosative stress resistance in *C. jejuni*. To fully understand the role of this enzyme *in vivo*, a further characterization of its catalytic properties would contribute to identifying the mechanism by which this enzyme supports *C. jejuni* virulence.

### Sulfite Dehydrogenases and Related Enzymes

Although better known as an antimicrobial preservative in food and beverages, sulfite is also present in the human body as a byproduct of amino-acid degradation that takes place mostly in the liver, while in serum, sulfite exists at micromolar levels in a protein-bound form that is less toxic (Ji et al., 1995; Lester, 1995; Mitsuhashi et al., 2005). Elevated serum sulfite levels have been observed in an LPS-stimulated rat model (Mitsuhashi et al., 2005) and also in patients with pneumonia or chronic renal failure (Kajiyama et al., 2000; Mitsuhashi et al., 2004), suggesting a pro-inflammatory activity of this molecule. Sulfite can also act as a signaling molecule in human cells, where it has been shown to promote proliferation of lymphocytes, as well as upregulation of reactive oxygen species (ROS) production in neutrophils, either directly or indirectly, via activation of alveolar macrophages to produce factors that stimulate neutrophils (Pettit et al., 1991; Beck-Speier et al., 1993; Mitsuhashi et al., 1998, 2005).

Interestingly, in response to LPS treatment, human neutrophils can also produce sulfite, at estimated local concentrations of 5–6 μM, likely to trigger an inflammatory response (Mitsuhashi et al., 2005). However, these physiological concentrations are unlikely to have significant bactericidal effects, as 0.96 mM sulfite was required to inhibit growth of *Helicobacter pylori in vitro* (Hawrylik et al., 1994), and at least 2 mM sulfite was needed to inhibit growth of *Lactobacillus* spp. and *Streptococcus* spp. (Irwin et al., 2017).

Despite the relatively low physiological concentrations of sulfite in at least the human host, a *C. jejuni* enzyme homologous to characterized SOEs (Figure 4) was shown to be required for successful infection of the Caco-2 human intestinal cell line. The *C. jejuni* sulfite dehydrogenase (SDH) has two components, the catalytic protein Cj0005, and a *c*-type cytochrome, Cj0004 (Myers and Kelly, 2005; Tareen et al., 2011). A Δ*cj0004c* strain showed reduced respiration on sulfite, while a Δ*cj0005c* strain exhibited a growth defect on sulfite-containing medium (Myers and Kelly, 2005; Tareen et al., 2011), indicating that these proteins are genuinely involved in *C. jejuni* sulfite oxidation. The Δ*cj0005c* mutant also showed decreased motility, as well as down-regulation of flagellar biosynthesis- and autoagglutination-associated genes that are important for virulence (Tareen et al., 2011), but the mechanisms underlying these additional physiological effects are unknown. At present, this appears to be the only direct evidence that SOEs can support host-cell colonization.

## DMSO Reductase Family Enzymes in Pathogens

Unlike the XO and SO family enzymes, enzymes of the DMSOR family occur uniquely in prokaryotes. They are functionally versatile and able to convert a diverse range of sulfur and nitrogen compounds such as DMSO, tetrathionate, trimethylamine N-oxide (TMAO), or nitrate, making many of them directly relevant to global sulfur and nitrogen cycles (McDevitt et al., 2002; Kappler and Schäfer, 2014; Leimkühler and Iobbi-Nivol, 2016). Additionally, enzymes of this family contribute to the detoxification of arsenic and selenium compounds, and degradation of aromatic compounds such as ethylbenzene (Hille et al., 2014).

**Figure 5:**
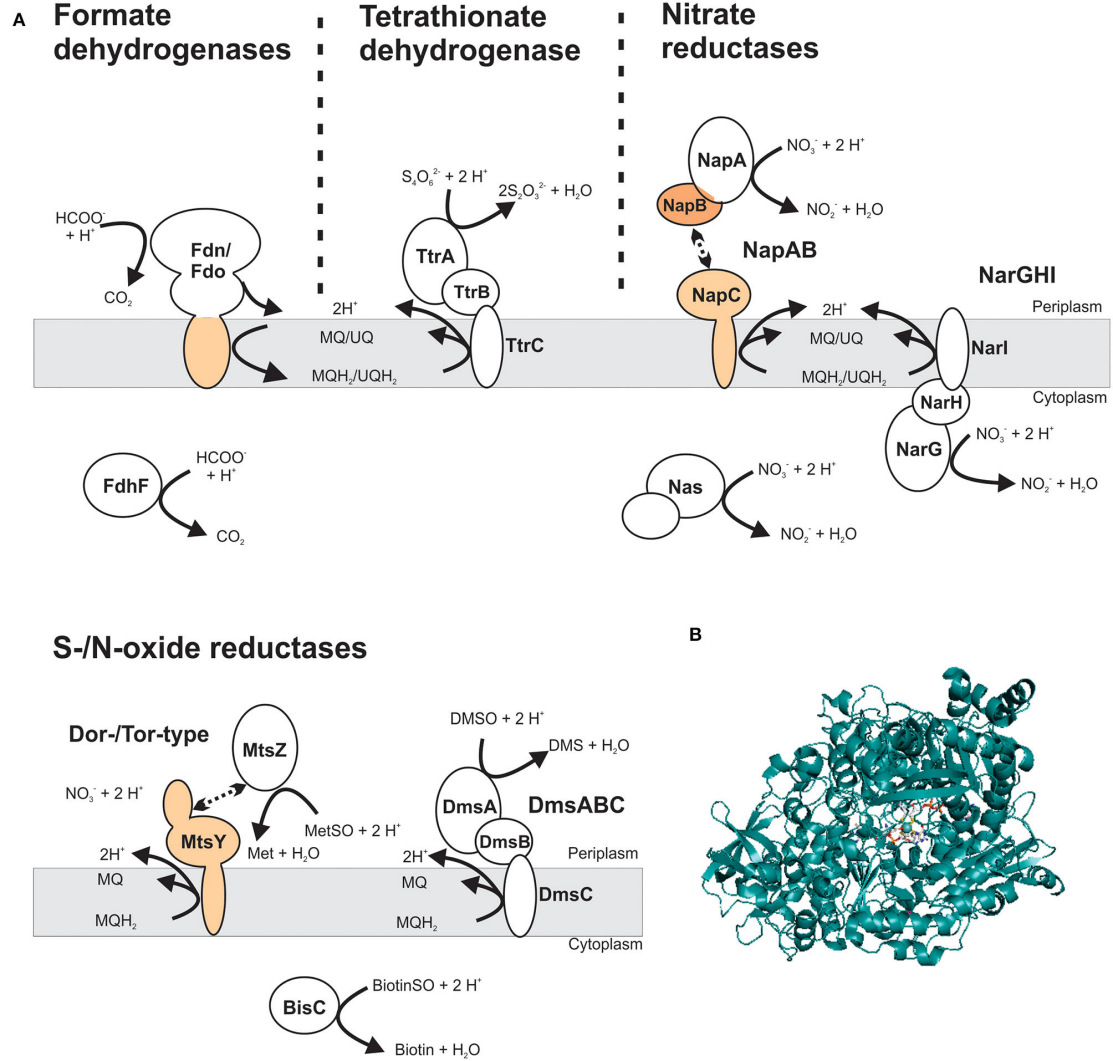
Structure and subcellular location of DMSOR family enzyme that support virulence in different bacterial pathogens. **(A)** Schematic representation of enzyme structures, showing both membrane-bound and soluble Mo-enzyme complexes. The most common structure for DMSOR family enzymes is heterotrimeric (αβγ), consisting of a Mo-containing subunit; an iron-sulfur protein and a membrane-integral subunit that may or may not bind hemes. Additionally, some enzymes that consist of a soluble Mo-binding subunit or a protein complex that includes the catalytic subunit that interacts transiently with a heme-binding membrane-integral cytochrome. Gray – Mo-binding subunit; orange – heme-binding subunits. MQ – menaquinone; MQH_2_ – menaquinol; UQ – ubiquinone; UQH_2_ – ubiquinol. **(B)** Crystal structure of NarG, the catalytic subunit of the respiratory nitrate reductase from Escherichia coli (PDB: 1R27) with the Mo-bisPGD cofactor shown in stick form. Colors: white – carbon, blue – nitrogen, orange – phosphor, red – oxygen, yellow – sulfur, light teal– molybdenum, protein backbone – teal.

DMSOR family enzymes very commonly have a heterotrimeric structure, consisting of a Mo-containing catalytic subunit, an iron-sulfur protein that transfers electrons to the catalytic subunit, and a membrane anchor subunit that connects the other redox centers to the cellular quinone pool (Figure 5) (Rothery et al., 2008; Grimaldi et al., 2013; Magalon et al., 2017). These heterotrimeric complexes can face either the bacterial cytoplasm or the periplasm (Grimaldi et al., 2013; Magalon et al., 2017). In some cases, two-subunit enzymes are also found and typically consist of a Mo-binding catalytic protein, or a multi-subunit catalytic protein complex (e.g., NapAB nitrate reductase), that interacts transiently with a membrane-bound, electron transfer subunit (Figure 5) (Arnoux et al., 2003; Grimaldi et al., 2013). All DMSOR family enzymes contain a Mo-bisPGD cofactor (Figure 5), and the four dithiolene sulfur atoms of the two cofactor molecules coordinate the Mo atom, together with an oxo- or a sulfido-group and an amino acid ligand (Johnson et al., 1990; Leimkühler et al., 2011; Leimkühler and Iobbi-Nivol, 2016; Kaufmann et al., 2018; Leimkühler, 2020). Different amino acid ligands including serine, cysteine, selenocysteine, and aspartate have been identified in DMSOR enzymes and their distribution largely coincides with three major phylogenetic groups identified in various studies: type I enzymes (e.g., NapAB nitrate reductase and FdnGHI formate dehydrogenase) contain a cysteine or selenocysteine ligand; type II enzymes, such as the heterotrimeric NarGHI nitrate reductase, the DmsABC DMSO reductase and the EbdABC ethylbenzene dehydrogenase, have an aspartate amino acid ligand; and the type III Dor/Tor-type S- and N-oxide reductases typically have a serine amino acid ligand (McDevitt et al., 2002; Grimaldi et al., 2013; Kappler and Schäfer, 2014; Wells et al., 2020). The AioAB arsenite oxidase is an outlier in this classification system, because of the absence of an amino acid ligand to the Mo; however, the AioAB amino acid sequence is most closely related to type I enzymes (McEwan et al., 2002; Warelow et al., 2013). Crystal structures as well as functional and spectroscopic properties are available for many of these enzymes and have been reviewed previously (Rothery et al., 2008; Magalon et al., 2011, 2017; Gonzalez et al., 2013; Grimaldi et al., 2013; Hille et al., 2014; Wells et al., 2020).

Many DMSOR family enzymes play an essential role in the bacterial respiratory chain as alternative terminal reductases that support anaerobic energy generation, or as dehydrogenases that donate electrons to the respiratory chain (Unden et al., 2014). Anaerobic respiration provides a fitness advantage to bacteria in low oxygen environments, including during interactions with a host, where oxygen depletion is common during host tissue inflammation (Winter et al., 2013a,b; Hughes et al., 2017).

### S- and/or N-oxide Reductases

S- and/or N-oxide reductases are a common type of DMSOR enzymes and occur in the structurally distinct type II and type III enzyme groups (Grimaldi et al., 2013). Both types of reductases have been implicated in supporting bacterial virulence (Baltes et al., 2003; Denkel et al., 2013; Dhouib et al., 2016). The heterotrimeric, membrane-bound DmsABC S/N-oxide reductase is a type II enzyme, and evidence for its involvement in bacterial virulence is currently mixed. DmsABC has been shown to be required for virulence in the pig pathogen *Actinobacillus pleuropneumoniae* (Baltes et al., 2003), but no mechanistic insights were provided beyond evidence of reduced survival of a Δ*dmsA* strain in a model of porcine pleuropneumonia. In contrast, in *E. coli*, DmsABC did not appear to be essential for colonization of the mouse gut (Jones et al., 2011; Hughes et al., 2017).

**Figure 6:**
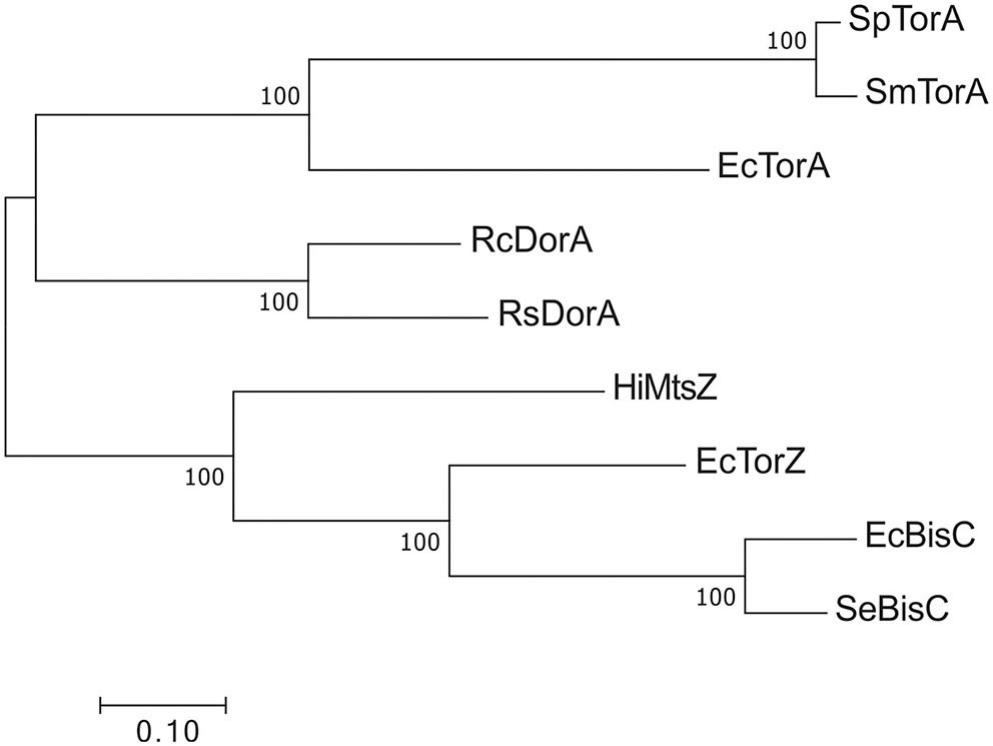
Phylogenetic relationships of Dor/Tor-type S- and N-oxide reductases of the DMSOR family. Sequences were aligned using MUSCLE in MEGA7 (Kumar et al., 2016) and phylogenetic relationships determined using the maximum-likelihood method. Robustness testing used bootstrapping (500 cycles), bootstrap values above 80 are shown near relevant nodes. All sequences were downloaded from the NCBI protein database. Grouping was well-supported by the bootstrap values. Sp TorA, *Shewanella putrefaciens* (WP_086903686.1); SmTorA, *Shewanella massilia* (WP_115334087.1); RcDorA, *Rhodobacter capsulatus* (WP_013068552.1); RsDorA, *Rhodobacter sphaeroides* (WP_011338998.1); HiMtsZ, *Haemophilus influenzae* (WP_0 108 69026.1); Se BisC, *Salmonella enterica* (WP_183053592.1); EcTorA, *Escherichia coli* (WP_001063164.1); EcTorZ (WP_000176781.1); EcBisC (WP_000013974.1).

By contrast, for type III S-/N-oxide reductases several studies have detailed a role in bacterial fitness and virulence (Denkel et al., 2013; Dhouib et al., 2016). Phylogenetically, the Dor/Tor enzymes form at least two groups (Figure 6) (Dhouib et al., 2016): the first group comprises the DorA DMSORs (Mouncey et al., 1997; Shaw et al., 1999) and the TorA TMAO reductases (Mejean et al., 1994; Dos Santos et al., 1998), both of which enable anaerobic respiration with their respective substrates; representatives of the other lineage are the BisC biotin sulfoxide reductase (Ezraty et al., 2005) and TorZ S/N-oxide reductase (Gon et al., 2000) from *E. coli* and the MtsZ methionine sulfoxide reductase from *H. influenzae* (Dhouib et al., 2016) (Figures 5, 6). The proposed substrates of BisC and MtsZ, biotin sulfoxide (BSO) and MetSO, can form following exposure of biotin or methionine to reactive oxygen species (ROS) produced by immune cells such as neutrophils and macrophages at sites of infections (Pattison and Davies, 2001; Ray et al., 2012; Kim et al., 2014). The ability to reverse sulfoxidation of amino acids and vitamins is important for bacterial survival, as the oxidative damage renders these molecules biologically inactive (Levine et al., 2000; Zempleni et al., 2009).

Only one enzyme of the DorA/TorA lineage, the TorA TMAO reductase from *E. coli*, has been studied for its relevance during infection, and it was dispensable for mouse intestinal colonization (Jones et al., 2011). In contrast, enzymes of the second lineage within the Dor/Tor enzyme group, such as the BisC biotin sulfoxide reductase from *S. enterica* serovar Typhimurium and the MtsZ methionine sulfoxide reductase from *H. influenzae*, have been shown to be important for bacterial virulence (Denkel et al., 2013; Dhouib et al., 2016).

BisC was originally described as a biotin sulfoxide reductase (del Campillo-Campbell and Campbell, 1982), but later studies in both *S*. Typhimurium and *E. coli* showed that this enzyme also has MetSO reductase activity and is essential for MetSO- and BSO-dependent growth in methionine or biotin auxotrophic strains (Ezraty et al., 2005; Denkel et al., 2013). In *S*. Typhimurium, inactivation of *bisC* resulted in growth defects in the presence of H_2_O_2_ and attenuation of virulence in macrophages and during intraperitoneal infections in mice (Denkel et al., 2013). Interestingly, the increased *in vitro* sensitivity to H_2_O_2_ of the *S*. Typhimurium Δ*bisC* strain was linked to the MetSO reductase activity of BisC, while attenuation of virulence in the infection models was caused by the loss of BSO reductase activity. The MetSO reductase activity of BisC was not essential for this function (Denkel et al., 2013). This may be due to the important role of biotin as a cofactor for enzymes with essential cellular functions such as acetyl-CoA carboxylase that plays a key role in fatty acid biosynthesis and is a known antimicrobial target (Freiberg et al., 2004). BisC is an unusual DMSOR family enzyme, as it is not encoded in an operon with electron-donating proteins, and the physiological electron donor for BisC is still unknown (Ezraty et al., 2005), but will likely provide further insights into mechanistic aspects and physiological functions of this enzyme.

While the MetSO reductase activity of BisC is dispensable for virulence in *S*. Typhimurium, a methionine sulfoxide reductase, the periplasmic MtsZ enzyme from the respiratory pathogen *H. influenzae*, was shown to be important for bacterial virulence (Dhouib et al., 2016). Within the BisC/TorZ/MtsZ lineage, MtsZ and related enzymes form a distinct phylogenetic clade (Dhouib et al., 2016), suggesting that MtsZ may have a different physiological role to the *E. coli* TorZ or BisC. In keeping with this, kinetic data showed that the *K*_M_ of MtsZ for BSO was in the millimolar range, making this activity unlikely to have physiological relevance, while that for MetSO was in the micromolar range (Dhouib et al., 2016). Like BisC, MtsZ only reduced free MetSO and was unable to repair protein-bound MetSO, but an obvious difference between the two enzymes is that MtsZ is linked to the *H. influenzae* respiratory chain, connecting it to redox balancing and energy generation in oxygen-limited niches (Dhouib et al., 2016). In *H. influenzae*, loss of MtsZ reduced biofilm formation and in-biofilm survival, and survival of the MtsZ mutant strains was attenuated in both human tissue cells and a mouse lung infection model (Dhouib et al., 2016). MtsZ was proposed to mediate redox balancing by reducing MetSO to methionine, which at the same time increases the availability of this amino acid as a nutrient.

However, not all enzymes in the BisC/TorZ/MtsZ lineage appear to affect in-host survival of bacterial pathogens. In *C. jejuni*, mutation in *cj0264*, which encodes an enzyme related to the BisC/TorZ/MtsZ group of S/N-oxide reductases, did not lead to a defect in a chicken caecum colonization model (Weingarten et al., 2008), and similarly, a *torZ* mutation in *E. coli* did not alter its ability to colonize the mouse intestine (Jones et al., 2011). As the physiological roles of these enzymes are currently unknown, it may be possible that, under other conditions, these enzymes are relevant for bacterial fitness, which may include other infection models. What is striking is the modulation of the substrate spectrum in enzymes of the MtsZ/BisC/TorZ group toward S-oxides found in the host; which has been demonstrated to correlate with specific and differential physiological functions during infection especially for BisC from *S*. Typhimurium.

### Nitrate Reductases

Nitrate is another compound that is readily converted by molybdoenzymes. It occurs naturally in terrestrial and aqueous environments and is used as a food additive to prevent bacterial spoilage (Gassara et al., 2016; Ma et al., 2018). At sites of infection, nitrate forms following reaction of nitric oxide with oxygen, with nitrite and peroxynitrite being other NO oxidation products, that together cause nitrosative stress in cells (Lundberg et al., 1994; Nathan and Shiloh, 2000; Bogdan, 2001; Enocksson et al., 2004). Nitrate can be used by many bacteria, including pathogens as an alternative terminal electron acceptor for anaerobic respiration, and in several cases, this mode of anaerobic energy generation has been linked to bacterial virulence and fitness in the host (Jones et al., 2007; Lopez et al., 2012, 2015; Spees et al., 2013; Winter et al., 2013b).

There are three types of prokaryotic nitrate reductases (NRs): the membrane-bound, cytoplasm-facing NarGHI respiratory NR and the periplasmic NapAB NR both enable nitrate-dependent anaerobic respiration, while the assimilatory NasA NR enables the use of nitrate as a nitrogen source (Goldman et al., 1994; Moreno-Vivián et al., 1999; Potter et al., 1999; Richardson et al., 2001; Sparacino-Watkins et al., 2014) (Figure 5). In addition to enabling anaerobic respiration with nitrate, NapAB has also been proposed to have roles in redox balancing and growth under nitrate-limited conditions (Potter et al., 1999). In *E. coli*, expression of both *nar* and *nap* operons is strongly induced during anaerobiosis, and *nar* expression is further enhanced when nitrate is present (Stewart, 1982; Grove et al., 1996; Wang et al., 1999). In contrast, oxygen status does not seem to affect expression of the *nas* operon; instead, and in keeping with its role as an assimilatory enzyme, its expression is induced by nitrate and repressed by ammonium (Ramos et al., 1993; Goldman et al., 1994).

While all three types of NRs are present in pathogenic bacteria (Jeter et al., 1984; Cali et al., 1989), roles in bacterial virulence have only been identified for Nar- and Nap-type NRs (Spector et al., 1999; Weber et al., 2000; Kohler et al., 2002; Jones et al., 2007; Van Alst et al., 2007; Weingarten et al., 2008; Tan et al., 2010; Kassem et al., 2012; Winter et al., 2013b; Lopez et al., 2015; Martín-Rodríguez et al., 2020).

Dissimilatory NRs in the enteric pathogens *E. coli* and *S*. Typhimurium are particularly well studied (Lundberg et al., 1994; Enocksson et al., 2004). Both *E. coli* and *S*. Typhimurium contain two Nar-type NRs, NarGHI and NarZYW, and a Nap-type NR, NapAB. Of the three NRs, NarG is the major contributor to dissimilatory nitrate reduction, although NapA also plays a part in nitrate reduction (Potter et al., 1999, 2000; Stewart et al., 2002; Gates et al., 2003; Martín-Rodríguez et al., 2020). Interestingly, NarZ seemed to contribute little to nitrate reduction and anaerobic growth on nitrate, and is repressed by anaerobiosis, even in the presence of nitrate (Iobbi-Nivol et al., 1987, 1990; Chang et al., 1999; Spector et al., 1999; Stewart et al., 2002).

In *E. coli*, NarG is the only nitrate reductase required for successful colonization of the mouse intestine, while NapA and NarZ are non-essential (Jones et al., 2007). However, some synergy between the different NR systems appears to exist, as the colonization defect observed for the Δ*narG* strain was more pronounced in both a Δ*narG* Δ*narZ* double mutant and a Δ*narG* Δ*napA* Δ*narZ* triple mutant (Jones et al., 2007). The *E. coli* triple NR mutant was strongly outcompeted by the WT strain during colonization of healthy and inflamed mouse gut environments (Jones et al., 2007; Spees et al., 2013; Winter et al., 2013b), and in a rat urinary tract infection model (Martín-Rodríguez et al., 2020). This documents that nitrate reduction confers a growth advantage to pathogenic *E. coli* strains and supports effective host colonization.

NarG is also the more important dissimilatory NR for virulence in the cystic-fibrosis-associated, opportunistic pathogen *P. aeruginosa*, where NarGHI, NapAB, and NasA NRs have been identified. Mutations in *P. aeruginosa narG* led to growth defects in cystic fibrosis sputum medium, reduced swarming ability *in vitro* and decreased virulence in *C. elegans* infections (Palmer et al., 2007; Van Alst et al., 2007), while a Δ*napA* mutant displayed wild-type phenotypes (Van Alst et al., 2007). The swarming defect was suggested to be attributable to a reduced level of rhamnolipid production, a swarming-facilitating biosurfactant that is only produced when exogenous nitrate is depleted (Van Alst et al., 2007). A loss of NarG also reduced biofilm formation and in-biofilm survival of *P. aeruginosa* (Van Alst et al., 2007). In the brucellosis-causing opportunistic human pathogen *Brucella suis*, where NarGHI is the only respiratory NR, a Δ*narG* strain was attenuated in human macrophages (Kohler et al., 2002), but NarGHI appeared to be dispensable for mouse infections (Loisel-Meyer et al., 2006).

However, NarG is not always a major virulence determinant, and in *S*. Typhimurium it was not required for virulence in a mouse model of colitis (Lopez et al., 2015). Instead, NapA was necessary for wild type-level virulence of *S*. Typhimurium in the mouse colitis model, and a Δ*narZ* mutant also showed somewhat reduced fitness in this infection model (Lopez et al., 2012, 2015). NarZ appears to have additional roles in *S*. Typhimurium, where *narZ* expression was induced upon interaction with tissue cells, and a Δ*narZ* mutant also showed significantly reduced virulence in a mouse oral infection model (Spector et al., 1999).

In *C. jejuni*, where NapA is the only NR encoded in the genome, NapA also appeared to be essential for *in vitro* resistance to H_2_O_2_ and colonization of the chicken caecum (Weingarten et al., 2008; Kassem et al., 2012; Liu et al., 2012).

NRs are also found in prominent non-Gram-negative pathogens. Both *Mtb* and *Mycobacterium bovis* contain two NR homologs, NarG and NarX, of which only NarG has nitrate reductase activity while the cellular function of NarX, a “fused-domain NR” with amino acid sequence similarity to the subunits of NarGHI, remains unclear (Hutter and Dick, 1999; Sohaskey and Wayne, 2003).

NarG-mediated nitrate reductase activity in *Mtb* increases during hypoxic conditions (Sohaskey and Wayne, 2003), and has been proposed as a marker for the transition from aerobic growth to non-replicating persistence of *Mtb* e.g., in granulomas (Wayne and Hayes, 1998). *In vitro*, exogenous nitrate, which would serve as a substrate for NRs, protected *Mtb* from radical nitrogen species and acid stress (Tan et al., 2010). Exogenous nitrate also enhanced survival of wild-type *Mtb* during sudden anaerobiosis *in vitro*, but not when oxygen was gradually depleted (Sohaskey, 2008), which suggests that the NarG NR plays a role in maintaining redox balance. Despite showing a loss of nitrate-dependent, anaerobic respiration and reduced survival *in vitro* (Aly et al., 2006; Malm et al., 2009), an *Mtb* Δ*narG* mutant exhibited wild type-level survival in a mouse lung infection model and primary human macrophages under low oxygen tension (Aly et al., 2006; Cunningham-Bussel et al., 2013), but was cleared more readily within infected macrophages by the anti-tuberculosis drug, isoniazid (Cunningham-Bussel et al., 2013). In contrast, NarG was essential for *M. bovis* infection of SCID mice and a loss of NarG also reduced persistence in BALB/c mice (Weber et al., 2000; Fritz et al., 2002).

Although studies of *Mtb* Δ*narX* mutant strains have not been reported, in *Mtb* clinical isolates, *narX* expression was observed in granuloma samples, and both *narX* and *narG* were induced in pericavity and distant lung samples, indicating a role for both enzymes during infection (Fenhalls et al., 2002; Rachman et al., 2006). This observation matches the elevated levels of NarX and NarG detected in the proteome of *Mtb* in a guinea pig infection model (Kruh et al., 2010).

In summary, dissimilatory nitrate reduction underpins fitness and virulence in many bacterial pathogens; however, the exact physiological roles and enzymes involved in nitrate reduction differ significantly between species, and various NR homologs exist in bacteria for which the exact physiological functions still need to be determined.

### Tetrathionate Reductases

In mammalian host organisms, the sulfur compound tetrathionate (S_4_${\text{O}}_{6}^{2-}$) forms in inflamed gut environments, where H_2_S is produced by the microbiota and converted to thiosulfate (S_2_${\text{O}}_{3}^{2-}$), by the action of mitochondria (Hildebrandt and Grieshaber, 2008). Thiosulfate can then be oxidized to tetrathionate by reactive oxygen species (Winter et al., 2010). Several different types of enzymes catalyze the reduction of tetrathionate, such as the membrane-bound, heterotrimeric TtrABC tetrathionate reductase (Hinojosa-Leon et al., 1986; Hensel et al., 1999), the octaheme tetrathionate reductases found in *Shewanella* spp. (Mowat et al., 2004), or thiosulfate dehydrogenases in, for example, *C. jejuni* (Liu et al., 2013). Of these three enzymes, only TtrABC is a molybdoenzyme (Hinojosa-Leon et al., 1986) (Figure 5), and the role of this enzyme in tetrathionate reduction was first discovered in *S. enterica* serovar Typhimurium (Knox et al., 1943). Tetrathionate reduction has since been shown to occur in other bacterial pathogens such as *P. aeruginosa, S. typhi*, and *Citrobacter freundii*, while *E. coli* and *Klebsiella pneumoniae* lack this activity (Barrett and Clark, 1987).

In *S*. Typhimurium, expression of the *ttr* operon is induced by anaerobiosis and the presence of tetrathionate (Hensel et al., 1999), but repressed by nitrate (Lopez et al., 2012), with the TtrSR two-component system as a major regulator of *ttr* expression (Hensel et al., 1999). A functional TtrABC complex was required for growth in tetrathionate-containing medium (Hensel et al., 1999), as well as for Aer-mediated energy taxis toward tetrathionate, likely because TtrABC modulates the redox state of *S*. Typhimurium (Rivera-Chávez et al., 2013).

An *S*. Typhimurium Δ*ttrA* mutant strain was outcompeted by the wild-type strain in both mouse and bovine gut colitis infection models, but not during infection of a healthy gut (Winter et al., 2010). In fact, the inflamed gut was the only body niche where the Δ*ttrA* strain showed reduced virulence compared to the wild-type strain, possibly due to the absence of tetrathionate in other body niches (Winter et al., 2010).

Unlike several of the other Mo enzymes discussed above, TtrABC thus confers a highly niche-specific fitness advantage to bacteria that is associated specifically with an inflamed gut. Confirming this advantage in other bacteria that harbor this enzyme, especially those that infect other body niches such as *P. aeruginosa*, would add further insights into the physiological roles of tetrathionate reductases.

### Formate Dehydrogenases

Formate dehydrogenases convert formate to CO_2_, and unlike the enzymes discussed previously in this section, they feed electrons into the cellular quinone pool rather than re-oxidizing them. The substrate of these enzymes, formate, is a product of serine catabolism in mammalian cells, but can also result from microbial fermentation in the GI tract (Pietzke et al., 2020). Once produced, formate can enter the circulation and is used in various key metabolic pathways, including nucleotide synthesis (Pietzke et al., 2020). Formate may also play a role during infections, as a 2-fold increase of formate concentration (at millimolar level) correlated with increased inflammation in the mouse gut (Hughes et al., 2017).

In bacteria, formate is converted to CO_2_ by one of three types of molybdenum-containing formate dehydrogenases (FDHs) (Figure 5). The two membrane-integral, periplasm-facing formate dehydrogenases, FDH-O (encoded by the *fdo* operon), and FDH-N (encoded by the *fdn* operon) (Figure 5), contribute to both aerobic and anaerobic respiration with formate as the electron donor (Ruiz-Herrera and DeMoss, 1969; Yamamoto and Ishimoto, 1977; Unden et al., 2014). Expression of *fdnG* is induced predominantly during anaerobiosis, when nitrate is present (Darwin et al., 1997; Wang and Gunsalus, 2003), while *fdoG* is expressed constitutively at low levels regardless of oxygen status (Abaibou et al., 1995; Benoit et al., 1998). The cytoplasmic FDH-H (*fdhF*) is a component of the formate hydrogen lyase complex that disproportionates formate into CO_2_ and H_2_ and is involved in redox balancing during fermentative growth (McDowall et al., 2014; Pinske and Sawers, 2016). In keeping with this role, expression of *fdhF* in *E. coli* is induced under anaerobic condition in the presence of formate, while oxygen and nitrate act as repressors (Birkmann and Bock, 1989; Rossmann et al., 1991; Wang and Gunsalus, 2003). Interestingly, some bacterial FDHs have no strict functional requirement for Mo as the catalytic metal center: the *C. jejuni* FDH, a distant relative of the respiratory FDH-N and FDH-O enzymes, is active with either a Mo- or W-bound cofactor; however, the same is not true for the *E. coli* FDHs, which are strictly molybdenum-dependent (Enoch and Lester, 1972; Andreesen and Ljungdahl, 1973; Taveirne et al., 2009; Niks and Hille, 2019).

Similar to what was reported above for the TtrABC tetrathionate reductase, and in keeping with increased formate production in the inflamed mouse gut, *E. coli* FDHs enhance bacterial survival in an inflamed gut, but are not required in the absence of inflammation (Hughes et al., 2017). Expression of both *fdnG* and *fdoG* in *E. coli* was induced during colonization of an inflamed mouse gut (Hughes et al., 2017). Single-gene knockout mutants revealed that FDH-N is a major contributor to *E. coli* fitness in a mouse colitis model, while FDH-O played a minor role (Hughes et al., 2017). This observation matches the increased expression of *fdnG* during anaerobiosis and may also be connected to the fact that nitrate, generated during inflammation, induces *fdnG* expression (Darwin et al., 1997; Wang and Gunsalus, 2003). However, the cellular roles of FDH enzymes vary, and in *Yersinia pestis*, a FDH-H mutation did not attenuate virulence in a mouse infection model (Pradel et al., 2014).

In contrast, FdhA, an enzyme distantly related to FDH-N from *E. coli*, supported virulence of *C. jejuni* in human and chicken cell line infections and chicken caecum colonization (Weerakoon et al., 2009; Kassem et al., 2012; Pryjma et al., 2012). In addition to respiratory chain-linked FDHs, there is also evidence that the cytoplasmic FDH-H can provide a fitness advantage, for example, to the human pathobiont *Aggregatibacter actinomycetemcomitans* in a murine abscess infection model (Jorth et al., 2013).

However, the role of FDHs in bacterial virulence is complex, and in *Shigella flexneri* Δ*fdnG* and Δ*fdoG* mutant strains even showed increased virulence in infections of a human intestinal cell line. This was attributed to the increase in formate concentrations in *S. flexneri* cells lacking functional FDHs, and the fact that in this bacterium, formate controls virulence gene expression (Koestler et al., 2018). Thus, in *S. flexneri*, FDHs are negative modulators of bacterial virulence, while in *E. coli* and *C. jejuni*, FDHs are positive modulators of bacterial fitness in the host (Koestler et al., 2018). Formate-based regulation of virulence factor expression was also observed in *S. enterica*, where formate upregulated the expressions of *sipC*, a gene encoding a type III secretion system effector protein (Lara-Tejero and Galán, 2009), and two major regulators in the *Salmonella* pathogenicity island 1, leading to enhanced virulence in the mouse gut (Huang et al., 2008). While infection data for *S. enterica* FDH mutant strains has not been reported, formate degradation might also be a negative regulator of virulence gene expression in *S. enterica*, but this needs to be investigated.

It thus appears that there are two distinct roles for FDHs in supporting or modulating bacterial virulence. The first role is in supporting preferred modes of energy generation, either by respiration or during fermentative growth, while the second appears to consist in controlling of the concentrations of formate in bacteria where formate acts as molecular signal that enhances expression of virulence-associated genes.

## Outlook

Mo enzymes from all three major families clearly have the potential to play important roles in supporting virulence and bacterial fitness in human pathogens, despite the fact that phylogenetic analyses indicate that these enzymes are not required in selected groups of bacterial pathogens that have undergone extreme adaptation of life in the host environment, such as the Chlamydiae.

While enzymes from the xanthine oxidase family are the least studied in this regard, and only CO dehydrogenases appear to have a possible role in bacterial virulence, for both the sulfite oxidase family and DMSO reductase family enzymes, there is clear evidence for the involvement of sulfite dehydrogenases, S/N-oxides reductases, nitrate reductases and formate dehydrogenases in supporting virulence in bacteria as diverse as *E. coli, S. enterica, C. jejuni, Mtb*, and *H. influenzae*.

**Figure 7:**
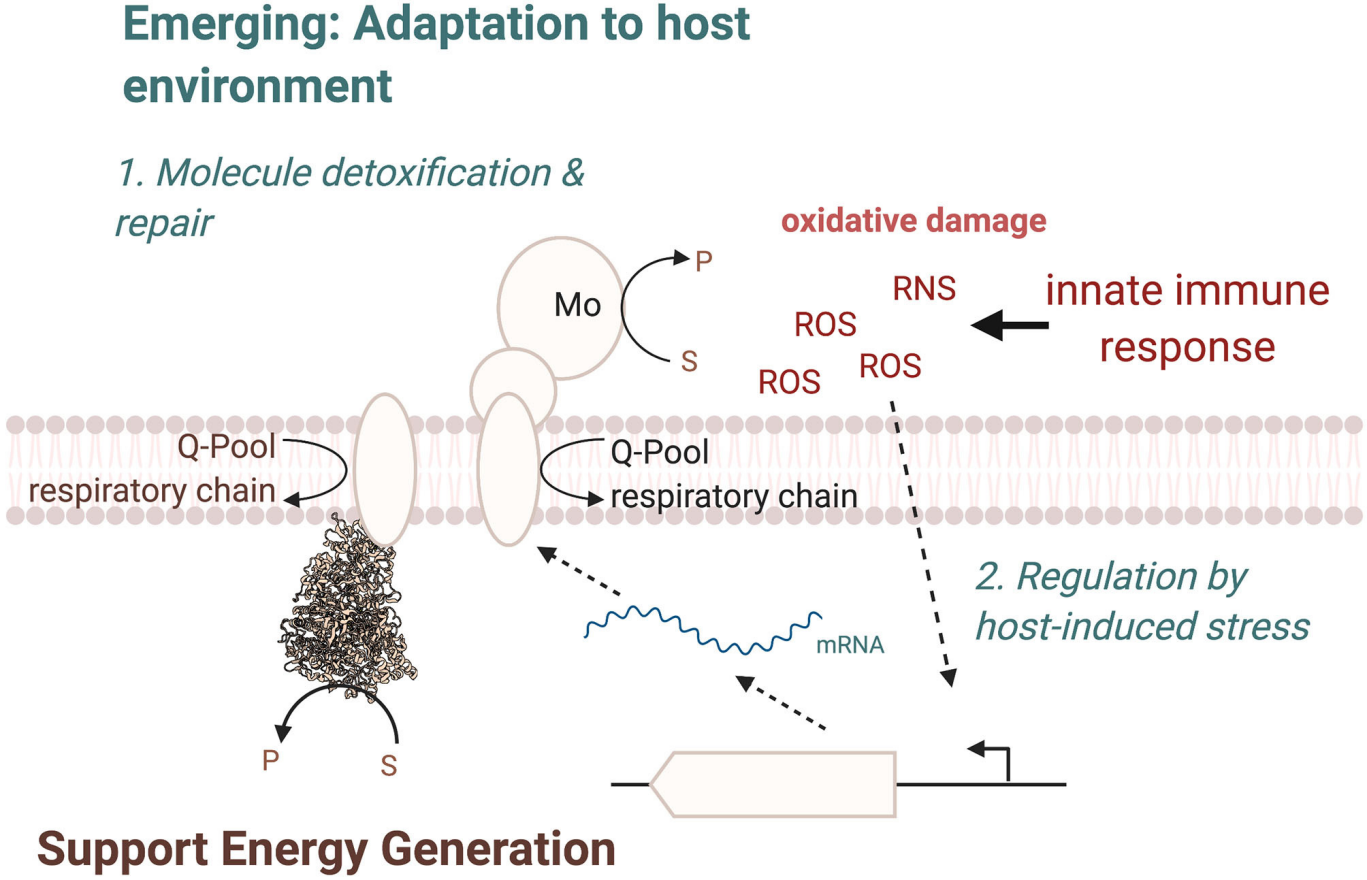
Overview of cellular functions of Mo-enzymes in bacterial pathogens. The three main functions are in energy generation, enabling optimal use of the metabolic network, and the two emerging functions of converting substrates that only occur in the human host and changes in the enzyme regulatory circuits, where Mo-enzyme expression is induced by molecules produced by the host organisms during infection. ROS, Reactive Oxygen Species; RNS, Reactive Nitrogen Species.

In most cases, the physiological roles of these enzymes appear to be very similar to those already identified for many molybdoenzymes, namely, in facilitating adaptation of the bacterial pathogen to its environment (Figure 7), by supporting energy generation and enabling the use or detoxification of compounds present in the host during inflammation. In this way, nitrate and tetrathionate reductases support anaerobic respiration in *E. coli, C. jejuni*, or *S*. Typhimurium (Jones et al., 2007; Weingarten et al., 2008; Winter et al., 2010, 2013b; Kassem et al., 2012; Liu et al., 2012; Lopez et al., 2012, 2015; Spees et al., 2013), while other enzymes such as FDH-H can be important for optimal fermentative growth (Jorth et al., 2013). While this physiological role is not surprising, the resulting fitness defects generally manifest themselves in a reduced ability of the pathogen to survive in the host environment, which highlights the importance of an optimally poised energy generating system for successful bacterial pathogenesis. The specific physiological roles of the Mo enzymes appear to be closely linked to the preferred growth modes of each bacterial pathogen, and in some cases, enzymes that are essential for in-host fitness of one pathogen are expendable in another organism, where the metabolic network has different functional requirements (Figure 7).

A second key consideration are the substrates that are converted by the Mo enzymes involved in bacterial virulence. Where the type of substrate has not changed during evolution, as is the case e.g., for nitrate or carbon monoxide, the source of the compound has changed and nitrate, for example, is predominantly produced from NO, while CO or sulfite are present because they act as signaling molecules or antimicrobials in the host (Figure 7). In other cases, notably the S- and N-oxide reductases, while the general reaction catalyzed is again similar to what has been identified in environmental bacteria such as the phototrophic *Rhodobacter* sp., the type of preferred substrate has changed. Instead of DMSO, which can occur in lake and marine environments (Kappler and Schäfer, 2014), the enzymes now preferentially convert sulfoxides and possible also N-oxides that are found in the human body, such as MetSO and BSO, both of which have been shown to be substrates converted by enzymes that are required for wild type-level virulence in different bacteria.

Thus, while S- and N-oxide reductases still have roles in supporting redox balancing and supporting anaerobic respiration, a subtle change in substrate preference appears to be occurring as a result of adaptation of bacteria to the host environment. To identify additional Mo enzymes that have the potential to support in-host fitness of bacterial pathogens, the presence of suitable substrates at sites of infection and in body niches that are colonized should be considered, as well as the possibility that some adjustment to the documented enzyme substrate preferences may have occurred.

A final point that should be considered when investigating the role of Mo enzymes in bacterial pathogens is the regulation of these enzymes in the pathogens (Figure 7). A well-established pattern of regulation for Mo enzymes is induction of expression in the presence of their substrate, which is often paired with increased expression in the absence of oxygen, which is in keeping with the known oxygen sensitivity of the Mo-PPT cofactor and the roles of many enzymes in anaerobic energy generation; this has been observed for nitrate and tetrathionate reductases as well as formate dehydrogenases. However, in some instances, a specific adaptation of the regulation of expression to the host environment seems to have occurred. As an example, expression of the MsrP methionine sulfoxide reductase is induced in response to exposure of the bacteria to HOCl and possibly other ROS, which are produced by the host during infection, rather than by the presence of the enzyme's substrate (Gennaris et al., 2015). This is a pathogen-specific adaptation in the way expression of MsrP is regulated and it seems likely that similar adaptations may have occurred for other Mo enzymes in pathogenic bacteria.

Finally, while there are many studies on Mo enzymes found in pathogenic *E. coli, S. enterica, C. jejuni*, and even *Mtb*, how Mo enzymes support survival of priority pathogens such as the ESKAPE pathogens or WHO priority pathogens is unclear at present. Many of these pathogens contain several Mo enzymes, including many of those that already have been shown to support bacterial virulence (Table 2), making them interesting targets for further research. Additionally, the majority of Mo enzymes that are relevant to bacterial pathogenicity belong to groups that are unique to prokaryotes, and thus could be promising targets for the development of new antimicrobial agents.

**Table 2:** Occurrence of mononuclear molybdenum enzymes in ESKAPE pathogens.

**Enzyme family**	**Enzyme(s)**	***E*^a^**	***S*^b^**	***K*^c^**	***A*^d^**	***P*^e^**	***E*^f^**
Xanthine oxidase family	XDH	No	No	Yes	Yes	Yes	Yes
	CODH	No	No	No	No	Yes	No
Sulfite oxidase family	MsrP/YedY-like enzymes	No	No	Yes	Yes	Yes	Yes
	SDH	No	No	No	No	No	No
DMSO reductase family	Dms-type S/N-oxide reductase	No	Yes	Yes	Yes	Yes	Yes
	Dor/Tor-type S/N-oxide reductase	No	Yes	Yes	Yes	Yes	Yes
	Dissimilatory NR (Nap)	No	Yes	Yes	Yes	Yes	Yes
	Dissimilatory NR (Nar)	No	Yes	Yes	Yes	Yes	Yes
	Assimilatory NR	No	Yes	Yes	No	Yes	Yes
	Tetrathionate reductase (Ttr)	No	No	Yes	No	Yes	No
	Respiratory FDH (Fdo/Fdn)	No	Yes	Yes	No	Yes	Yes
	Fermentative FDH	No	Yes	Yes	Yes	Yes	Yes

*The presence of the enzymes was determined using BLASTP searches with representative Mo-enzyme amino acid sequences as input against the genomes of Enterococcus faecium (taxid:1352), Staphylococcus aureus (taxid:1280), Klebsiella pneumoniae (taxid:573), Acinetobacter baumannii (taxid:470), Pseudomonas aeruginosa (taxid:287) or Enterobacter species (taxid:547) in NCBI. Input sequences were: FdnG (WP_011310337.1); FdoG (WP_010723259.1); FdhF (WP_012767779.1); TtrA (WP_023197956.1); NapA (WP_063120336.1); NasA (WP_124037922.1); NarG (WP_000032935.1); NarZ (WP_063123841.1); TorZ (WP_187225196.1); TorA (WP_185171730.1); DmsA (WP_062859481.1); BisC (WP_137594927.1); SorA (WP_013168043.1); MsrP (WP_185168873.1); XdhA (WP_136858672.1); CoxL (WP_013913730.1)*.

a *Enterococcus faecium*.

b *Staphylococcus aureus*.

c *Klebsiella pneumoniae*.

d *Acinetobacter baumannii*.

e *Pseudomonas aeruginosa*.

f *Enterobacter species*.

*Yes, enzyme is supposedly present in this organism; No, enzyme is not present in this organism; XDH, xanthine dehydrogenase; CODH, carbon monoxide dehydrogenase; SDH, sulfite dehydrogenase; NR, nitrate reductase; FDH, formate dehydrogenase*.

## Author Contributions

QZ carried out the literature review, drafting, editing of all manuscript sections, carried out data analyses, and created tables and figures. BK provided critical analysis of the manuscript and help with the preparation of figures. UK provided help with the initial conception, structuring of the manuscript, contributed to manuscript writing, and to the generation of figures. All authors contributed to the article and approved the submitted version.

## Conflict of Interest

The authors declare that the research was conducted in the absence of any commercial or financial relationships that could be construed as a potential conflict of interest.
